# CLINICIAN-PERCEIVED CHALLENGES OF OSTEOPOROSIS CARE IN AN INTEGRATED HEALTHCARE DELIVERY SYSTEM

**DOI:** 10.1093/geroni/igz038.2775

**Published:** 2019-11-08

**Authors:** Aaron T Seaman, Melissa J Steffen, Karla Miller, Samantha Solimeo

**Affiliations:** 1 Iowa City VA Healthcare System, Iowa City, Iowa, United States; 2 Veterans Rural Health Resource Center Central Region Center for Access and Delivery Research and Evaluation Primary Care Analytics Team, Iowa City, Iowa, United States; 3 Veterans Rural Health Resource Center Western Region Department of Internal Medicine, Rheumatology Section Division of Rheumatology; University of Utah School of Medicine, Salt Lake City, Utah, United States; 4 Veterans Rural Health Resource Center Central Region, Center for Access and Delivery Research and Evaluation Primary Care Analytics Team, Iowa City, Iowa, United States

## Abstract

The burden of osteoporosis, both on the health care system and individuals, is high. Despite this, a high percentage of patients with or at risk of osteoporosis are not identified, screened and treated appropriately. Delivering osteoporosis care to at-risk patients is complicated by a fractured health care delivery system. In this presentation, we present data from interviews with VA clinicians in order to identify challenges of osteoporosis care within the VA health care system. While clinicians reported initiating a range of bone health care delivery interventions, they identified challenges that inhibited long-term sustainability: 1) low prioritization of bone health among national and facility leadership; 2) fragmentation of clinical responsibility and care delivery; and 3) barriers endemic to the osteoporosis care delivery system. Our results indicate that, even within an integrated health care delivery system, significant coordination challenges exist.

